# New Hampshire1The following paragraph contained in the original bill was eliminated in the Senate: “The New Hampshire Veterinary Medical Association shall furnish to the Governor, by the first day of January each year, a list containing five names, from which the yearly appointment shall be made.”

**Published:** 1901-05

**Authors:** 


### LEGISLATION.

### NEW HAMPSHIRE.[1]

The following act, restricting the practice of veterinary medicine in New Hampshire, has passed the Legislature, and was signed by Governor Jordan on March 8th, when it became a law.

HOUSE BILL 259. NEW DRAFT. INTRODUCED BY SMALL, OF ROCHESTER.

An act to restrict the practice of veterinary medicine to graduates of recognized medical schools.

*Be it enacted by the Senate and House of Representatives in general court convened :*

Section 1. No person shall use the name or title of veterinary surgeon or V.S. in this State after the first day of June, 1901, unless such person shall be registered in accordance with this act.

Sec. 2. Within sixty days after the passage of this act the governor shall appoint a board of three veterinary examiners, so appointed that the term of office of one member shall expire each year during the first three years, and that the members thereafter appointed shall hold office three years, or until successors are appointed and qualified.[1] Each veterinary examiner shall receive a commission of his appointment from the governor, and shall file forthwith with the Secretary of State the constitutional oath of office.

Sec. 3. From the fees provided in this act the board of veterinary examiners shall pay all proper expenses incurred by the provisions of this act.

Sec. 4. The board of veterinary examiners shall hold meetings for the purpose of registration and examination at such times and in such towns or cities as the board may determine. Examinations shall be in English, and embrace such subjects as materia medica, practical chemistry, physiology, anatomy, surgery, pathology. Applicants who shall pass the prescribed examination shall be registered and granted the right to use the name or title of veterinary surgeon or V.S.

[1] The following paragraph contained in the original bill was eliminated in the Senate: "The New Hampshire Veterinary Medical Association shall furnish to the Governor, by the first day of January each year, a list containing five names, from which the yearly appointment shall be made."

Sec. 5. This act shall not apply to persons who at the time of the passage of this act are engaged in the practice of veterinary surgery or medicine.

Sec. 6. Any person who, after April 1, 1901, desires to practice veterinary surgery and medicine in this State, and who is a graduate of a lawfully constituted college or institution of veterinary science, shall be granted registration and entitled to use the name or title of veterinary surgeon or V.S. by paying to the board of veterinary examiners the sum of two dollars.

Sec. 7. Any person who, after April 1, 1901, not being a graduate as described in the preceding section, may desire registration, shall apply to the board of veterinary examiners for examinations as to his qualifications, and such person upon passing such examination shall be granted registration and entitled to use the name or title of veterinary surgeon or V.S. by paying to the board of veterinary examiners the sum of five dollars.

Sec. 8. The board of veterinary examiners shall keep a record book in which shall be entered the names of all persons who comply with the provisions of this act, and the said record book shall be open to public inspection.

Sec. 9. Any person who shall use the name or title of veterinary surgeon or V.S. in contravention to the provisions of this act shall be guilty of a misdemeanor, and on conviction thereof shall be fined for each offence the sum of fifty dollars.

Sec. 10. This act shall take effect.

